# Editorial

**Published:** 1840-05-16

**Authors:** 


### THE MEDICAL EXAMINER.

### PHILADELPHIA, MAY 16, 1840.

At the request of a number of subscribers of the Examiner, we commence the publication of a series of lectures on physical exploration, and on the diagnosis and treatment of the diseases of the chest. Experience abroad has shown most clearly that the requisite supply of original matter for a weekly cannot be obtained without either introducing a very large proportion of isolated cases, which the majority of readers scarcely look at, or of resorting to courses of lectures which are from time to time delivered both in various medical institutions, both public and private. If the latter are uninteresting to those readers who may have devoted much attention to the subject, they are useful to a great proportion who may have never made the department which forms the subject of the lectures an object of especial study, or who may derive more complete information upon the subject than they can readily obtain from the ordinary sources.

The lectures on the diseases of the chest will contain a complete course, as full as may be convenient: they will be afterwards published in a separate form for the convenience of those who may desire the work separate from the Examiner.

